# Immune cell patterns before and after neoadjuvant immune checkpoint blockade combined with chemoradiotherapy in locally advanced esophageal squamous cell carcinoma

**DOI:** 10.1186/s12885-024-12406-3

**Published:** 2024-05-27

**Authors:** Dan-Dan Zheng, Yu-Ying Li, Xiao-Yi Yuan, Jiang-Li Lu, Mei-Fang Zhang, Jia Fu, Chris Zhiyi Zhang

**Affiliations:** 1 MOE Key Laboratory of Tumor Molecular Biology and State Key Laboratory of Bioactive Molecules and Druggability Assessment, Institute of Life and Health Engineering, College of Life Science and Technology, Jinan University, Guangzhou, 510632 China; 2 Department of Pathology, Sun Yat-sen University Cancer Center, Guangzhou, 510060 China

**Keywords:** Esophageal squamous cell carcinomas, Neoadjuvant immune checkpoint blockade combined with chemoradiotherapy, Pathologic complete response, Macrophages, Immune landscape

## Abstract

**Background:**

Neoadjuvant immune checkpoint blockade (ICB) combined with chemoradiotherapy offers high pathologic complete response (pCR) rate for patients with locally advanced esophageal squamous cell carcinomas (ESCC). But the dynamic tumor immune microenvironment modulated by such neoadjuvant therapy remains unclear.

**Patients and methods:**

A total of 41 patients with locally advanced ESCC were recruited. All patients received neoadjuvant toripalimab combined with concurrent chemoradiotherapy. Matched pre- and post-treatment tissues were obtained for fluorescent multiplex immunohistochemistry (mIHC) and IHC analyses. The densities and spatial distributions of immune cells were determined by HALO modules. The differences of immune cell patterns before and after neoadjuvant treatment were investigated.

**Results:**

In the pre-treatment tissues, more stromal CD3 + FoxP3 + Tregs and CD86+/CD163 + macrophages were observed in patients with residual tumor existed in the resected lymph nodes (pN1), compared with patients with pCR. The majority of macrophages were distributed in close proximity to tumor nest in pN1 patients. In the post-treatment tissues, pCR patients had less CD86 + cell infiltration, whereas higher CD86 + cell density was significantly associated with higher tumor regression grades (TRG) in non-pCR patients. When comparing the paired pre- and post-treatment samples, heterogeneous therapy-associated immune cell patterns were found. Upon to the treatment, CD3 + T lymphocytes were slightly increased in pCR patients, but markedly decreased in non-pCR patients. In contrast, a noticeable increase and a less obvious decrease of CD86 + cell infiltration were respectively depicted in non-pCR and pCR patients. Furthermore, opposite trends of the treatment-induced alterations of CD8 + and CD15 + cell infiltrations were observed between pN0 and pN1 patients.

**Conclusions:**

Collectively, our data demonstrate a comprehensive picture of tumor immune landscape before and after neoadjuvant ICB combined with chemoradiotherapy in ESCC. The infiltration of CD86 + macrophage may serve as an unfavorable indicator for neoadjuvant toripalimab combined with chemoradiotherapy.

**Supplementary Information:**

The online version contains supplementary material available at 10.1186/s12885-024-12406-3.

## Introduction

Esophageal cancer (EC) is the eighth most common cancer and the seventh leading cause of cancer-related mortality worldwide [1]. Esophageal squamous cell carcinoma (ESCC), with a 5-year survival rate less than 25% for patients receiving surgery alone, accounts for more than 90% of EC cases in East Asia [2]. Both CROSS trial and NEOCRTEC5010 trials confirmed that neoadjuvant chemoradiotherapy (nCRT) followed by esophagectomy offered the great benefit of long-term survival over 10 years to patients with locally advanced ESCC [3, 4]. Neoadjuvant chemotherapy (nCT) followed by surgery showed a 3-year overall survival comparable with nCRT among patients with locally advanced ESCC [5]. Lately, a phase 1b trial using adebrelimab showed a pathological complete response (pCR) rate of 8% and 2-year OS of 92%, suggesting neoadjuvant anti-PD-L1 monotherapy as a therapeutic strategy for patients with resectable ESCC [6]. The NEOCRTEC1901 trial revealed that neoadjuvant PD-1 blockade (toripalimab) combined with concurrent chemoradiotherapy followed by surgery in resectable locally advanced ESCC helped to improve pCR rate up to 50% [7].

The efficacy of immunotherapy largely depends on the tumor microenvironment. Numerous studies have shown that the infiltrations of immune cells, such as T lymphocytes and macrophages, contributed to patients’ responses to immune checkpoint blockade (ICB) [8, 9]. Neoadjuvant therapies are able to modify the anti-tumor response of ICB by modulating the immune microenvironment phenotype [10]. For example, tumors with immune-enriched signature exhibited more pathological tumor regression [6]. More macrophage infiltrations predicted well response to neoadjuvant camrelizumab combined with chemotherapy [11]. Pre-existing T cells in tumor contributed to the well response to neoadjuvant anti-PD-L1 immunotherapy [6]. A CCR4/CCR6 chemokine-based model was considered useful to predict the benefits from neoadjuvant chemoradiotherapy combined with ICB therapy [12]. However, the comprehensive grasp of the immune landscape before and after neoadjuvant PD-1 blockade combined with chemoradiotherapy has not been explored.

In this study, we intended to elucidate the association between immune cell infiltration and the efficacy of neoadjuvant anti-PD-1 immunotherapy combined with chemoradiotherapy. We recruited 41 locally advanced ESCC patients who received neoadjuvant therapies to examine and compare the immune landscape before and after treatment. Our findings characterized the therapy-induced modulation of immune cell patterns and provided new insights for the development of neoadjuvant toripalimab combined with chemoradiotherapy.

## Methods and materials

### Patient recruitment and sample collection

A total of 44 patients with locally advanced ESCC in Sun Yat-sen University Cancer Center (SYSUCC) were recruited in this study. Patients clinically staged as T1-4aN1-3M0 or T34aN0M0 before treatment received neoadjuvant toripalimab combined with concurrent chemoradiotherapy (CRT) followed by surgery. Four cycles of weekly intravenous 50 mg/m^2^ paclitaxel and 25 mg/m^2^ cisplatin on days 1, 8, 15, 22, and two cycles of 240 mg toripalimab on days 1 and 22 were given to the patients. A total dose of 40.0 Gy was administered in 20 fractions of 2.0 Gy, five fractions per week, starting at the first day of the first cycle of chemotherapy. Surgery was scheduled in six to eight weeks after completion of neoadjuvant therapy. No adjuvant treatment was administered following esophagectomy. Final, 41 patients received R0 surgery. Pretreatment biopsies were collected from 35 patients, and post-treatment surgical samples were obtained from 41 patients. Clinicopathological features and follow-up information were also gathered. This study received approval from the ethics committee of Sun Yat-sen University Cancer Center, and all included patients provided written informed consent.

### Pathological analysis and definition

Pathological reports presented the tumor type and extension, proximal and distal resection margins, lymph nodes status and tumor regression grade (TRG) of patients. Accordingly, all of the 41 patients were characterized into two groups:

(I) Non-pCR was defined as pCR not being achieved. pN0 referred to the category of non-pCR in which no residual tumor existed in the resected lymph nodes; pN1 referred to the category of non-pCR in which residual tumor existed in the resected lymph nodes.
(II) pCR was defined as no evidence of residual tumor cells in the primary site and resected lymph nodes of the operative specimens;

In addition, based on the TRG scores, patients were divided into four groups:

(I) TRG0: No viable tumor cells, including lymph nodes.
(II) TRG1: Single or rare small groups of tumor cells.
(III) TRG2: Residual cancer with evident tumor regression but more than small groups of tumor cells.
(IV) TRG3: Extensive residual cancer with no evident tumor regression.

### Fluorescent multiplex immunohistochemistry (mIHC)

The formalin-fixed paraffin-embedded tissues of ESCC patients were cut into sections of 4-µm thickness and mounted on glass slides. Fluorescent multiplex immunohistochemistry (mIHC) was conducted using the PANO 5-plex IHC kit (Panovue, Being) in sequential staining cycles to explore the tumor immune microenvironment in ESCC patients before trentment. The slides were first deparaffinized in xylene and rehydrated in ethanol. Then, antigen retrieval was performed with the EDTA buffer (pH 9.0, ZhongShan Golden Bridge, Beijing) by microwave treatment (MWT) for 15 min. After incubation with blocking buffer (Panovue, Being) to block nonspecific binding, slides were incubated with the primary antibody at room temperature for 60 min. Then, the sections were washed using TBST buffer and incubated with the HRP-conjugated secondary antibody for 10 min before visualization using fluorophore based on tyramide signal amplification (TSA). After, MWT was repeated to remove the non-covalently bound antibodies and TSA complex. Slides were stained sequentially with the following antibodies and fluorescent dye, in order: anti-CD3/Opal-520, anti-CD8/Opal-570, anti-Foxp3/Opal-650, anti-Cytokeratin /Opal-480. TSA-stained slides were counterstained with DAPI for 10 min, coverslipped using the mounting media and finally imaged by Vectra Polaris Automated Pathology Imaging platform (Perkin Elmer, Waltham) at 20 x magnification. The second panel includes anti-CD86/Opal-520, anti-CD15/Opal-570, anti-CD163/Opal-650, anti-Cytokeratin/Opal-480; the third panel includes anti-CD8/Opal-520, anti-PD-1 /Opal-570, anti-PD-L1/Opal-650, anti-Cytokeratin/Opal-480.

### Immunohistochemistry (IHC)

IHC was used to evaluate the characterization of tumor infiltrating immune cells of ESCC patients after treatment based on manual immunohistochemistry, by staining with the following markers: CD3, CD8, CD15, CD86, CD163, FoxP3, PD-1 and PD-L1. Formalin-fixed paraffin-embedded tissues were cut into 4 μm sections and baked at 60 °C for 3 h, following by being dewaxed with xylene and rehydrated with gradient ethanol. Antigen retrieval was performed with the antigen retrieval solution (ZhongShan Golden Bridge, Beijing) in a pressure cooker, boiling for 2.5 min. The sections were then treated with 3% hydrogen peroxide to quench endogenous peroxidase activity, following by incubating with primary antibodies at 37 °C for 50 min. After three rounds of washing with PBS buffer, the tissue sections were incubated with anti-Rabbit or mouse HRP secondary antibody (Zhongshan Golden Bridge, Beijing) for 20 min, and washed with PBS buffer for another three times. DAB Chromogen substrates were added to the sections for staining, and the staining intensity was observed under the microscopy. The sections were scanned by an automatic slice scanning system (Leica Biosystems).

### Image analysis

mIHC and IHC images were analyzed using digital pathology analysis platform HALO (Indica Labs, Corrales). The researchers were blinded to follow-up details of patients during the staining, scanning and analysis period. For IHC, we first used the tissue classification algorithm in HALO to divide ESCC tissues into intratumor region and interstitial region according to the tissue and cell morphology. Then, we used the HALO® Multiplex IHC v3.1.4 algorithm to identify positive immune cells according to DAB staining status. For mIHC, we also first performed tissue classification on these images. We next applied HALO® Highplex FL v4.1.3 algorithm to identify different kinds of immune cells according to the cell morphological features and the pattern of fluorophore expression. After cell identification, we explored the spatial distribution of different kinds of immune cells via the proximity analysis algorithm of HALO.

### Statistical analysis

Computational and statistical analyses were performed using R software (Version 4.1.2) and GraphPad Prism software (Version 8.0.2). Cell density of the immune phenotypes was calculated per mm^2^ of tissues. Student’s t-test was used to compare immune cell characteristics between pCR and non-pCR group. Pearson correlation coefficient was used to analyze the correlation between the density of immune cell infiltration in the interstitial region and the stroma region.

## Results

### Characteristics of the patients and the overall study workflow

**Fig. 1:**
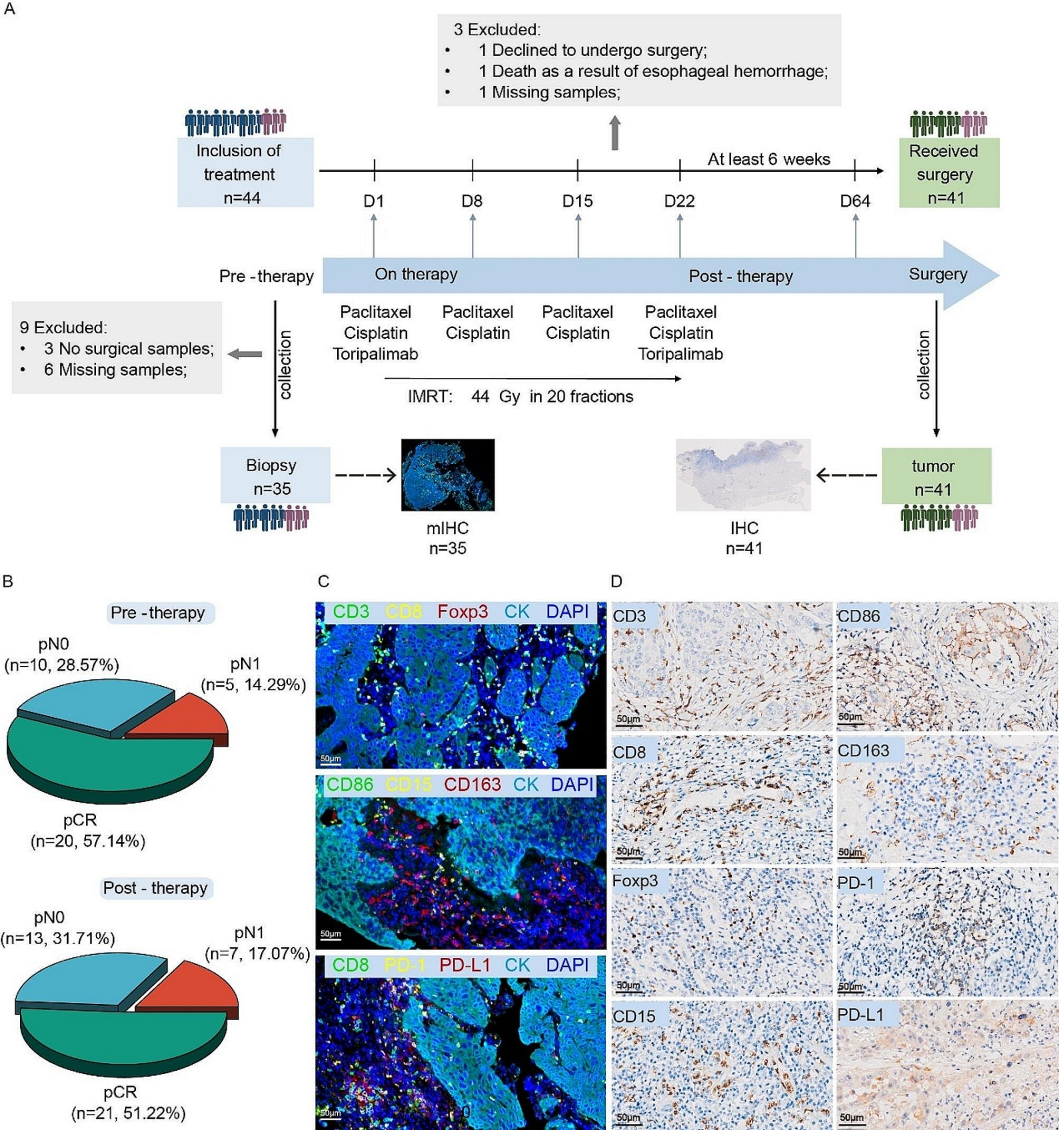
Study design and data generation. **(A)** Sample collection and data generation. A total of 41 patients were included in this study. Thirty-five pre-treatment biopsies and 41 post-surgery formalin-fixed and paraffin-embedded (FFPE) tissues were collected. **(B)** Pie charts showed the post-surgery pathological composition of patients with available pre-treatment biopsies or surgical tissues. **(C)** mIHC analysis was conducted to assess the tumor immune microenvironment (TIME) in pre-treatment tissues. Three panels of immune markers were used in our study. **(D)** Representative IHC images showed the expression of eight types of immune cell markers

A total of 44 patients with locally advanced ESCC were recruited and given toripalimab combined with neoadjuvant chemoradiotherapy (nCRT) in Sun Yat-sen University Cancer Center (SYSUCC). Among them, 41 patients, with a male/female ratio 32:9 and a median age of 60 years, received R0 resection. The TRG scores were as follows: TRG 0 (23/41, 56.10%), TRG 1 (10/41, 24.39%), and TRG 2 (8/41, 19.51%). Thirty-five pre-treatment biopsies and 41 post-surgery formalin-fixed and paraffin-embedded (FFPE) tissues were collected in our study. Fluorescent multiplex immunohistochemistry (mIHC) was performed on 35 biopsy tissues to reveal the immune cell infiltration before treatment. The immune patterns were determined by immunohistochemistry (IHC) in 41 postsurgical paraffin-embedded tissues (Fig. 1A).

According to the pathological examination reports, patients were divided into pCR, pN0 and pN1 groups. After pathologic evaluation of 41 patients, 21 patients achieved pCR, 13 patients achieved pN0 and seven patients were pN1 (Fig. 1B). Representative images of mIHC and IHC of eight immune cell markers (CD3, CD8, CD15, FoxP3, CD86, CD163, PD-1 and PD-L1) were shown in Fig. 1C&D.

### Stromal immune cell infiltrations in pretreatment tissues are associated with pathological response

**Fig. 2:**
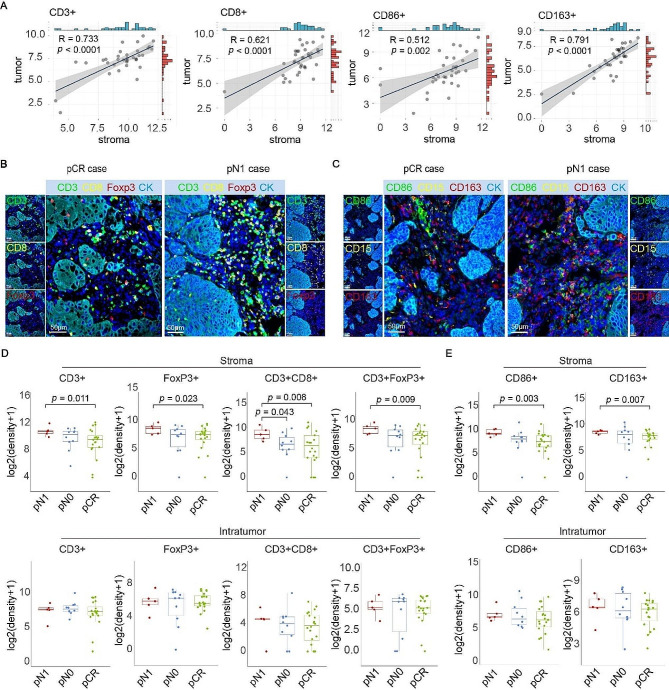
The immune landscape in the stromal region of pretreatment tissues show a correlation with postoperative pathological grading. **(A)** Pearson correlation coefficient was used to compare the correlations of CD3+, CD8+, CD86 + and CD163 + cell densities in the stroma and tumor areas of 35 cases. Cell density is represented as the number of cells per area (mm^2^). **(B)** Representative mIHC images showing the difference in CD3 + CD8+, CD3 + FoxP3 + cell infiltration in pN1 pre-treatment tissues. **(C)** Representative mIHC images showing the CD15+, CD86+, and CD163 + cell infiltrations in pCR and pN1 patients. **(D)** Comparison of CD3+, FoxP3+, CD3 + CD8+, and CD3 + FoxP3 + T lymphocytes infiltration in distinct regions among cases with pCR, pN0 and pN1. The infiltrations of T lymphocytes only in stroma of pretreatment tissues were associated with the pathological response. **(E)** The infiltration of CD86+, and CD163 + macrophages in different regions were compared among pCR, pN0 and pN1 group. The infiltrations of macrophages only in stroma of pretreatment tissues were associated with the pathological response. (pCR, *n* = 20; pN0, *n* = 10; pN1, *n* = 5. Statistical significance was determined using a T-test)

To characterize the immune landscape in pretreatment samples, we applied mIHC to analyze the immune cell composite in 35 biopsies. We firstly examined the densities of infiltrating immune cells in both intratumor and stromal areas. The results showed that T cells, macrophages, and granulocytes were mainly distributed in the stroma (Figure S1A). The infiltrations of those immune cells in stroma and intratumor were positively correlated (Fig. 2A; Figure S1B).

Next, we compared the infiltrations of T cells, macrophages and granulocytes in patients with different post-surgical pathological grades. T cell types were identified by mIHC, using antibodies of CD3 (all types of T cell), CD8 (cytotoxic T cell) and FoxP3 (regulatory T cell) (Fig. 2B), while macrophages and granulocytes distribution were determined by positive staining of CD86 (M1 macrophage), CD163 (M2 macrophage), and CD15 (granulocyte) (Fig. 2C). The mIHC results demonstrated that there was no significant difference in immune cell patterns between pCR and non-pCR patients (Figure S2A). For patients with different TRG scores, the densities of immune cells infiltrating in both intratumor and stroma remained unchanged (Figure S2B). However, for the pN1 patients who had the worst prognosis, there was more FoxP3 + and CD3 + FoxP3 + regulatory T cells (Tregs) in the stromal region (Fig. 2D). Stromal CD3 + CD8 + cells were more frequently presented in patients with pN1, compared with pN0 or pCR patients (Fig. 2D). Furthermore, the cell densities of both CD86 + and CD163 + macrophages in the stroma area of pN1 patients were much higher than those of pCR patients (Fig. 2E). Compared with pCR patients, the distributions of CD8+, CD15+, PD1 + and PD-L1 + lymphocytes in both intratumor and stroma of pN0 and pN1 patients were not significantly different (Figure S2C). Collectively, our data suggest the infiltrations of T lymphocytes and macrophages in stroma of pretreatment tissues were associated with the pathological response in patients receiving neoadjuvant toripalimab combined with chemoradiotherapy.

### Macrophage-enriched TME phenotype as potential unresponsive biomarker

**Fig. 3:**
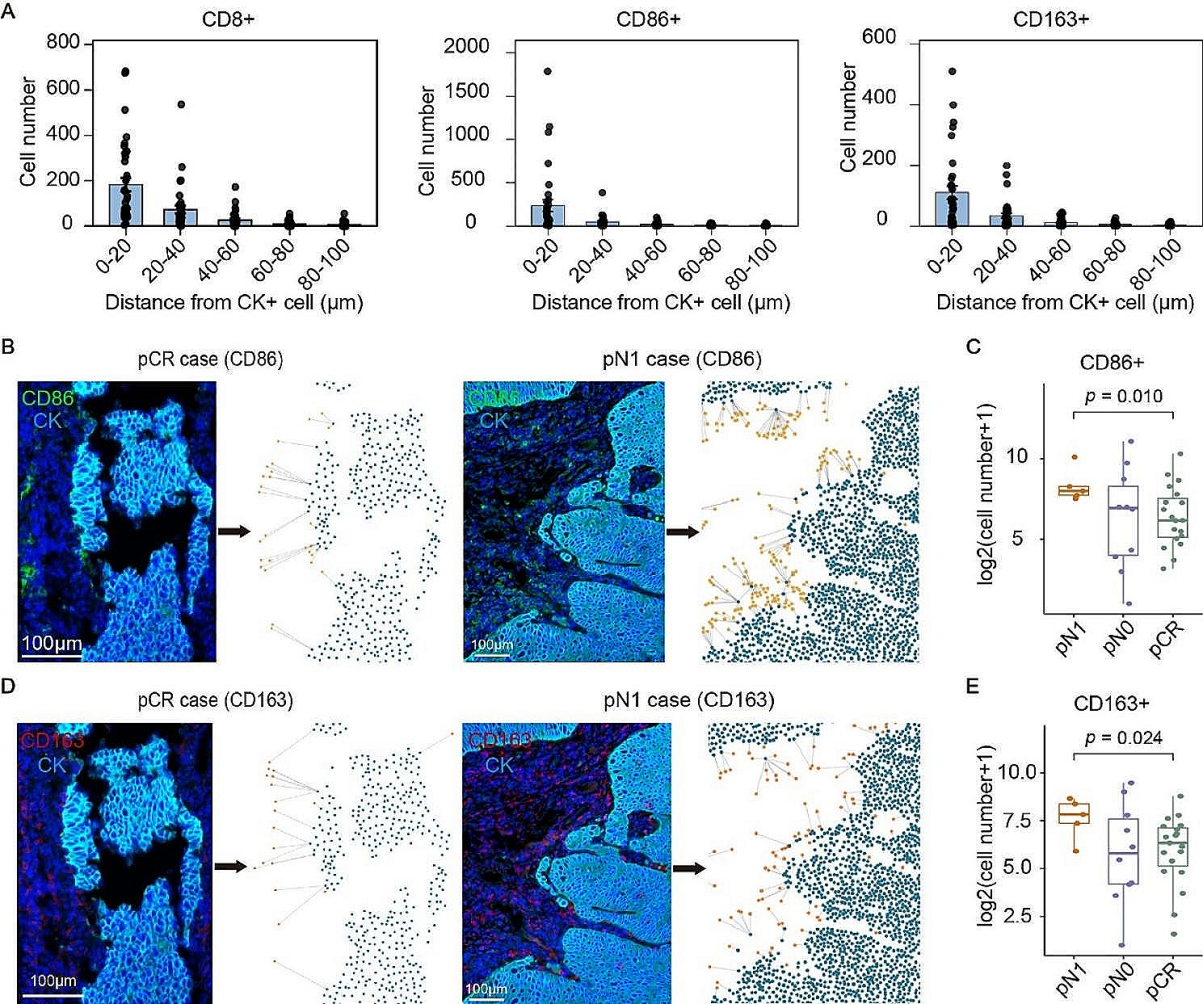
The spatial distributions of macrophages in pre-treatment tissues were related to the postoperative pathological grade. **(A)** The numbers of CD8+, CD86+, and CD163 + cells within the indicated distance to the CK + tumor cells were counted and presented by histogram. **(B)** Representative images showed the spatial distribution of CD86 + M1 macrophages within 40 μm to tumor cells in pre-treatment pCR and pN1 tissues. **(C)** Comparison of the quantity of infiltrating CD86 + M1 macrophages within a 40 μm proximity to tumor cells in pre-treatment pCR, pN0, and pN1 ESCC tissues. **(D)** Representative images showed the spatial distribution of CD163 + M2 macrophages within 40 μm to tumor cells in pre-treatment pCR and pN1 tissues. **(E)** Comparison of the quantity of infiltrating CD163 + M2 macrophages within a 40 μm proximity to tumor cells in pre-treatment pCR, pN0, and pN1 ESCC tissues. (pCR, *n* = 20; pN0, *n* = 10; pN1, *n* = 5. Statistical significance was determined using a T-test.)

The spatial distribution of tumor-infiltrating lymphocytes contributes to the antitumor immunity. We performed spatial analyses of immune cell distribution in pretreatment tissues, using the proximity analysis algorithm of HALO. The results showed that stromal immune cells were mainly distributed within the range of 0–40 μm to the CK + tumor cells (Fig. 3A; Figure S3A). Thus, we conducted a comparison of the quantity of immune cells infiltrating within 40 μm to cancer cells in pre-treatment tissues. A significant increase of CD86 + M1 (Fig. 3B, C) or CD163 + M2 (Fig. 3D, E) macrophage was found in pN1 patients, compared with pCR samples, indicating a macrophages-enriched TME phenotype in pN1 tissues. Nonetheless, no significant differences were detected in the spatial distribution of T lymphocytes or granulocytes between different postoperative pathological grading (Figure S3B). In addition, the spatial distance between PD-1 + and PD-L1 + cells was not related to the pathological grade (Figure S3C). These findings suggest the macrophage-enriched TME phenotype as a potential factor for unresponders.

### Macrophage infiltration in post-treatment tissues correlate with pathological outcome

**Fig. 4:**
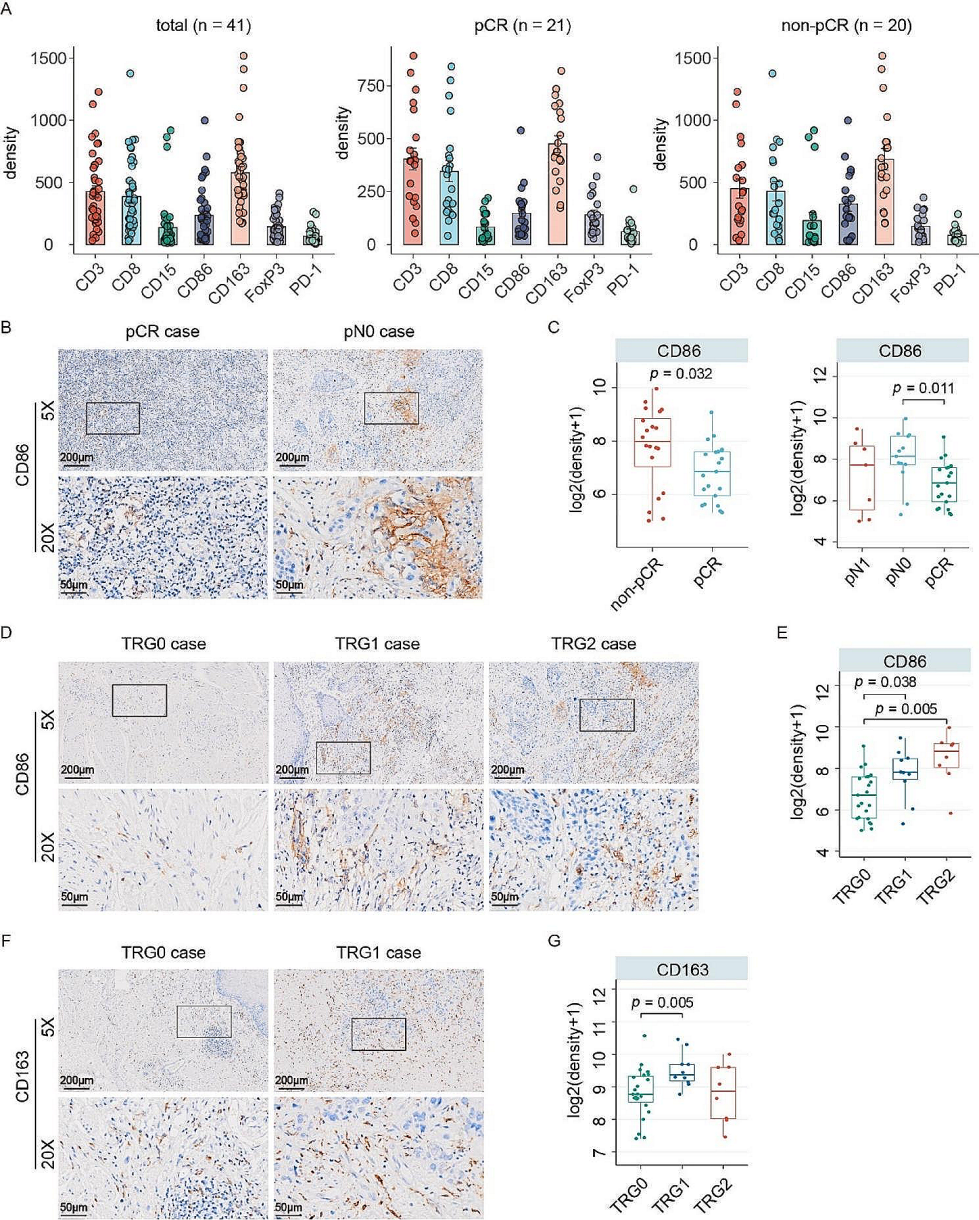
Clinical significance of macrophage infiltration in post-treatment tissues. **(A)** Immune cell compositions in post-surgical tissues of total, non-pCR and pCR patients were indicated. **(B)** Representative IHC images showed the distribution of CD86 + M1 macrophages infiltration in post-treatment pCR and pN0 tissues. **(C)** The densities of CD86 + M1 macrophages in patients with indicated pathological grades were compared. **(D)** Representative IHC images showed the distinction in CD86 + M1 macrophages infiltration among TRG0, TRG1 and TRG2 tissues. **(E)** The densities of CD86 + M1 macrophages in patients with different TRG scores were compared. **(F)** Representative IHC images showed the CD163 + M2 macrophages infiltrations in TRG0 and TRG1 cases. **(G)** The cell densities of CD163 + M2 macrophages in post-surgical samples were compared. (TRG0, *n* = 23; TRG1, *n* = 10; TRG2, *n* = 8; pCR, *n* = 21; pN0, *n* = 13; pN1, *n* = 7. Statistical significance was determined using a T-test.)

Understanding the immune cell landscape of post-treatment tissues in patients receiving neoadjuvant immunotherapy helps to improve the second-line strategy, especially for the unresponders. As there was no residual tumor in pCR samples, we evaluated the post-treatment immune pattern by quantifying the immune cell infiltrating in the entire pathological slide. The post-treatment cell densities of T lymphocytes, macrophages and granulocytes in total, non-pCR and pCR samples were presented in Fig. 4A. Compared with the pCR patients, non-pCR patients had more CD86 + M1 macrophages. We further compared the infiltration of CD86 + cells in pCR, pN0, and pN1 samples, the results showed that pN0 patients, but not pN1, had significantly higher CD86 + M1 macrophage infiltration than pCR samples (Fig. 4B&C). For the patients with different TRG scores, we found that the CD86 + M1 macrophage infiltration in TRG0 cases was significantly less than TRG1 and TRG2 cases (Fig. 4D&E). On the other hand, the CD163 + M2 macrophages infiltration increased in TRG1 cases but remained unchanged in TRG2 cases, compared with TRG0 (Fig. 4F&G). Nevertheless, the infiltration patterns of other tested immune cells in the post-treatment tissues were similar in patients with different pathological grades (Figure S4). Collectively, infiltration of CD86 + macrophage in post-treatment tissues may reduce the response to neoadjuvant immunotherapy combined with nCRT in ESCC.

### Dynamic evolution of the TME status in response to neoadjuvant therapy

**Fig. 5:**
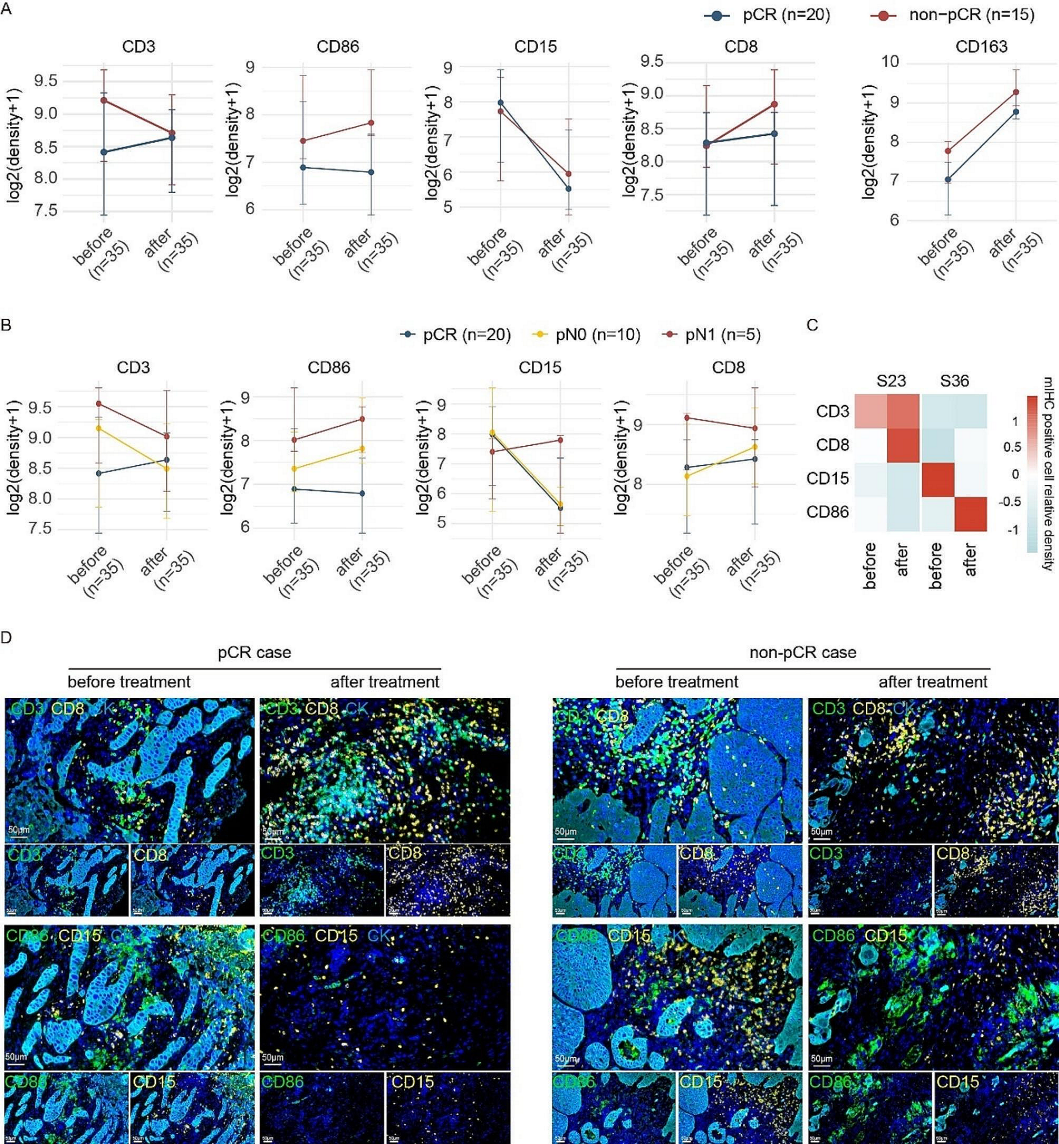
Dynamics of immune landscape before and after neoadjuvant toripalimab combined with chemoradiotherapy in ESCC. **(A)** Densities of CD3+, CD8+, CD15 + and CD86 + cells before and after treatment between the pCR and non-pCR samples were compared. Points represent median values, whereas whiskers show the upper and lower quantiles. **(B)** Densities of CD3+, CD8+, CD15 + and CD86 + cells before and after treatment among the pCR, pN0 and pN1 samples were compared. Points represent median values, whereas whiskers show the upper and lower quantiles. **(C)** The densities of immune cells in sample 23 (pCR) and sample 36 (non-pCR) in post-treatment tissues were determined by mIHC. The heatmap showed the related infiltrations in tissues before and after treatment. **(D)** Representative mIHC images demonstrated the alterations in CD3+, CD8+, CD15 + and CD86 + cell infiltration in pCR and non-pCR patients

Our data demonstrated that immune landscape varied in pre-treatment and post-treatment tissues. We next investigated the TME dynamics before and after neoadjuvant toripalimab combined with CRT. For T lymphocytes, the density of CD3 + cells in pre- and post-treatment tissues had opposite trends in pCR and non-pCR patients, whereas CD8 + cell infiltration increased in post-surgical tissues of both pCR and non-pCR patients with marked differences in the magnitude of changes (Fig. 5A). For macrophages, CD86 + cells in post-treatment tissues increased in non-pCR patients, but decreased in pCR patients, compared with those in pretreatment tissues (Fig. 5A). However, the evolution of CD163 + M2 macrophages and CD15 + granulocytes were quite similar (Fig. 5A). Significant reduction of PD-1 + cells and remarkable induction of PD-L1 + cells were depicted in post-treatment tissues (Figure S5A).

We next determined the treatment-induced evolution of immune pattern in non-pCR patients. The results showed that CD3+, FoxP3+, PD-1+, PD-L1+, CD86 + and CD163 + cells exhibited consistent alterations between pN0 and pN1 patients (Fig. 5B and S5B). For CD8 + and CD15 + cells, similar changes were observed in pN0 and pCR patients, but there was an opposite trend of evolution in pN1 patients (Fig. 5B).

To better demonstrate and verify the comparability of pre- and post-treatment immune landscape, the infiltrations of immune cells before and after treatment were examined by multiplex immunofluorescence (mIHC). S23 and S36 represented pCR and pN0 samples, respectively. The modulations of CD3 + T cells, CD8 + T cells, CD15 + granulocytes and CD86 + macrophage infiltrations in pre- and post-treatment were compared. The heatmap indicated similar alterations of CD8 + and CD15+, but opposite trends of CD86 + and CD3 + cells between pCR and pN0 patients (Fig. 5C). mIHC data demonstrated the enhanced infiltration of CD3 + and CD8 + T lymphocytes and the loss of CD15 + granulocytes and CD86 + macrophages in pCR patients (Fig. 5D).

## Discussion

Several clinical trials recommend neoadjuvant therapy for patients with locally advanced ESCC. Recently, the combination of anti-PD-1 antibody toripalimab and concurrent CRT as neoadjuvant therapy for locally advanced ESCC resulted in a pCR rate of 50%. Notably, patients achieving pCR exhibited a more favorable survival trend compared to those without [13, 14]. However, there is an unmet clinical need for biomarker-based precision immunotherapy. Substantial evidence demonstrates that radiotherapy and chemotherapy play an immunogenic role and suggests a correlation between ICB and outcome [15, 16]. Patients exhibiting specific immune landscape characteristics may derive enhanced benefits from the addition of neoadjuvant immunotherapy alongside chemoradiotherapy. Therefore, conducting a thorough comparison of the immune landscape patterns before and after the treatment of toripalimab combined with CRT helps to elucidate the potential connection between the immune landscape and the effectiveness of neoadjuvant immunotherapy and CRT. Our data demonstrated that pre-existing stromal macrophages and CD3 + FoxP3 + T cells were significantly associated with the residual tumor existed in the resected lymph nodes. The more infiltration of CD86 + in non-pCR patients, the more residual tumor in primary site. In addition, the dynamic evolution of immune cell pattern was correlated with pathological response.

The antitumor response to PD-1/PD-L1 blockade is generally induced by the activation of CD8 + T lymphocytes [13]. Studies on neoadjuvant immunotherapy in solid tumors have found that post-treatment clonal expansion of CD3 + and CD8 + T cells, along with tissue-resident macrophages, correlates with pathological response [17, 18]. nCRT is capable of inducing the significant infiltrations of CD3 + CD8 + T cells and CD86 + macrophages, probably resulting in improved efficacy of immunotherapy [19–21]. However, several studies reported that there were abundant bystander CD8 + T cells surrounding the tumor, suggesting that not all CD8 + T cells trigger the anti-tumor immunity [22, 23]. Furthermore, the heterogeneity of CD8 + cells of irresponsive to ICB treatment has also been documented. Exhausted CD8 + T cells expressing SPRY1 (CD8 + Tex-SPRY1), by inducing proinflammatory phenotype of macrophages, correlated with complete response to neoadjuvant PD-1 blockade in advanced ESCC [24]. Our data demonstrated that more CD3 + CD8 + and CD3 + FoxP3 + T cells were distributed in the stroma region of pN1 pretreatment tissues, compared with the pCR ones. However, spatial distribution analyses indicated no difference of CD3 + CD8 + or CD3 + FoxP3 + T cells infiltrating within 40 μm to cancer cells were found between pN1 and pCR patients. These data may suggest that T lymphocytes were mainly distributed in the stroma and far away from the tumor nest, whereas M1 macrophages mainly gathered around the edge of the tumor areas to form an immunosuppressive TME and restrict T cell migration into the tumor through long-lasting contact, which results in the poor response to neoadjuvant treatment in pN1 patients.

The role of macrophage infiltration in the prediction of response to neoadjuvant treatment in ESCC has been identified [11]. Elevated tumor-associated macrophages (TAM) density in ESCC is correlated with tumor progression and shorter survival. nCRT in ESCC markedly induced the infiltration of M2 macrophage [13]. Activities of both M1-and M2-related pathways decreased overall in macrophages from major responders but increased in those from minor responders after nCRT [16]. Single-cell RNA sequencing showed that CCR4 + CCR6 + M1 macrophages were attenuated by neoadjuvant therapies [12]. Difference of macrophage infiltration was also depicted in the post-treatment tissues in this study. More CD86 + M1, but not CD163 + M2 macrophage was detected in the non-pCR tissues after immunotherapy combined nCRT, compared with pCRs. Furthermore, non-pCR patients with higher TRG scores had more CD86 + cells infiltration. These findings indicated that CD86 + macrophage may serve as an unfavorable indicator for neoadjuvant toripalimab combined with chemoradiotherapy.

Neoadjuvant toripalimab combined with CRT resulted in the modulation of immune cell landscape. Increased CD163 + cells and PD-L1 + cells were observed in both pCR and non-pCR post-treatment tissues. CD86 + cells showed a slight decrease in pCR patients, but a marked increase in non-pCR patients. CD3 + and CD8 + T cells were remarkably decreased in pN1 patients, but were increased in pCR patients. These changes of immune landscape formed an immunosuppressed TME in non-pCR patients. Literatures have also reported modulation of the TME by neoadjuvant therapy in locally advanced ESCC, showing enhanced infiltrations of macrophages and PD-L1 + cells in non-pCR patients [25, 26]. Interestingly, the alterations of T cell and macrophage infiltration have been demonstrated in patients receiving nCRT alone or neoadjuvant immunotherapy alone [6, 13]. Neoadjuvant chemotherapy in gastric cancer patients induced dynamic changes in infiltration of CD8 + T cells and total macrophages, with high-density infiltration of FoxP3 + Tregs in the stromal region before treatment being associated with treatment non-response [27]. Neoadjuvant chemoradiotherapy induced overexpression of PD-L1 and significant infiltration of T cells and CD86 + macrophages in solid tumors [19–21]. Neoadjuvant immunotherapy in ESCC indicate that high-density infiltration of CD8 + SPRY1 + T cells enhances immunotherapy efficacy [24]. Studies on neoadjuvant immunotherapy in other solid tumors have found that post-treatment clonal expansion of CD3 + and CD8 + T cells, along with tissue-resident macrophages, were correlated with pathological response [17, 18]. Thus, future investigations are required to determine whether the dynamic changes of immune cell patterns in our study are caused by chemoradiotherapy or anti-PD-1 immunotherapy.

A limitation of this study is incompletion of all types of immune cells, such as NK cells and B lymphocytes. A single cell proteomics study indicated CD16 + NK cells were accumulated in the tumor primary sites after nCRT combined with ICB treatment [12]. Second, not all of the paired samples, especially for pN1 patients, was obtained. Pervious study showed the pN1 patients had the worst prognosis in the nCRT setting [28]. NEOCRTEC1901 trial showed nCRT combined with ICB treatment markedly reduced the residual tumor in lymph nodes [29]. Third, the data in this study need to be verified by a larger cohort.

In summary, immune landscape before and after neoadjuvant toripalimab combined with CRT in locally advanced ESCC has been investigated in this study. Our data indicate that immune cell infiltration interplayed with the treatment of neoadjuvant toripalimab combined with CRT, especially Tregs and CD86 + macrophages. The dynamic immune cell pattern was significantly modulated by neoadjuvant treatment. Our study therefore provides new insights for the development of neoadjuvant strategies for ESCC.

### Electronic supplementary material

Below is the link to the electronic supplementary material.

**Supplementary Material 1**: **Figure S1**. Infiltration of immune cells in pretreatment samples. (A) The Wilcoxon-rank sum test was used to compare the distribution of immune cells between tumor areas and stroma areas in each sample before treatment. (B) Pearson correlation coefficient was used to compare the correlation of FoxP3+, CD3 + CD8+, CD3 + FoxP3+, CD15+, PD-1+, CD8 + PD-1 + and PD-L1 + cell infiltration density in the stroma and tumor area. Cell density is represented as the number of cells per area (mm^2^) analyzed.

**Supplementary Material 2**: **Figure S2.** Comparison of immune cells infiltration density in pretreatment samples in different pathological grades. (A) The infiltration of immune cells in different regions were compared among pCR and non-pCR. (B) The infiltration of immune cells in different regions were compared among TRG0, TRG1 and TRG2. (C) The infiltration of CD8+, CD15+, PD-1, CD8 + PD-1 + and PD-L1 + cells in different regions were compared among pCR, pN0 and pN1. (Statistical significance was determined using a T-test.)

**Supplementary Material 3**: **Figure S3.** Spatial distribution of immune cells in pretreatment samples. (A) CD3+, FoxP3+, PD-1 + and CD15 + cells localization around the tumor areas and they infiltration into the tumor are expressed by the distance from CK + cells in µm. (B) Comparison of the quantity of infiltrating CD3+, CD8+, CD3 + CD8+, FoxP3+, CD3 + FoxP3+, CD15 + and PD-1 + cells within a 40 µm proximity to tumor cells in pre-treatment pCR, pN0, and pN1 ESCC tissues. (C) Comparison of the quantity of infiltrating PD-1 + T lymphocytes within a 40 µm proximity to PD-L1 + cells and CD8 + PD-1 + T lymphocytes within a 40 µm proximity to CK + PD-L1 + tumor cells in pre-treatment pCR, pN0, and pN1 ESCC tissues. (Statistical significance was determined using a T-test.)

**Supplementary Material 4**: **Figure S4.** Comparison of infiltration density of immune cells in post-treatment samples in different pathological grades. (A) The infiltration of immune cells in different regions were compared among pCR and non-pCR. (B) The infiltration of immune cells in different regions were compared among pCR, pN0 and pN1. (C) The infiltration of immune cells in different regions were compared among TRG0, TRG1 and TRG2. (Statistical significance was determined using a T-test.)

**Supplementary Material 5**: **Figure S5**. Changes in the immune landscape during treatment. (A) Densities of FoxP3+, PD-1 + and PD-L1 + cells before and after treatment between the pCR and non-pCR samples. (B) Densities of FoxP3+, CD163+, PD-1 + and PD-L1 + cells before and after treatment among the pCR, pN0 and pN1 samples. Points represent median values, whereas whiskers show the upper and lower quantiles.

## Data Availability

The data that support the findings of this study are available from the corresponding author upon reasonable request.
