# Supplementary figures and images for: Immune cell patterns before and after neoadjuvant immune checkpoint blockade combined with chemoradiotherapy in locally advanced esophageal squamous cell carcinoma
Source: BMC Cancer. 2024 May 27;24:649. doi: 10.1186/s12885-024-12406-3 (PMC11129487; doi:10.1186/s12885-024-12406-3)

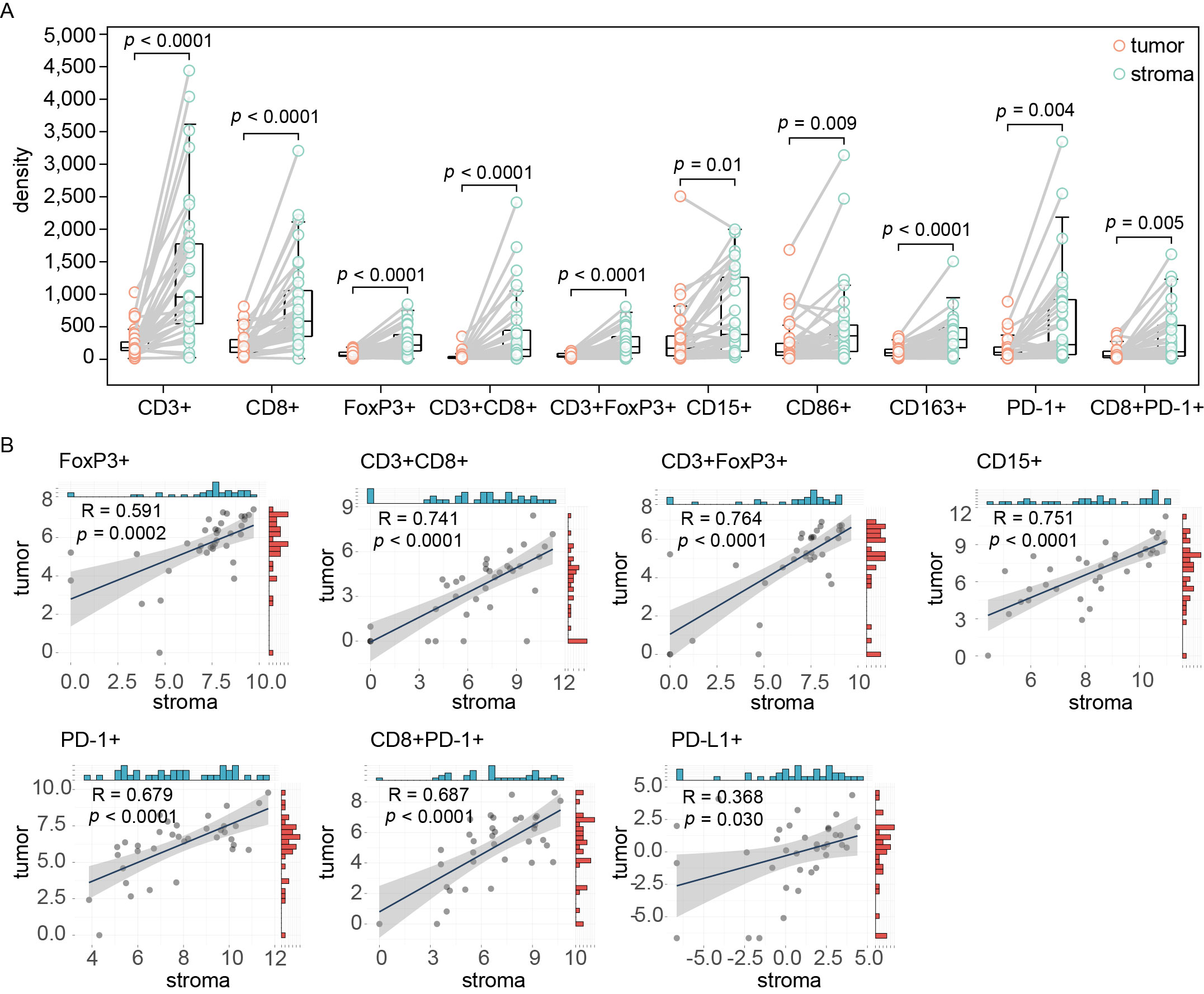

Supplement: Supplementary file 1 — Supplementary Material 1: Figure S1. Infiltration of immune cells in pretreatment samples. (A) The Wilcoxon-rank sum test was used to compare the distribution of immune cells between tumor areas and stroma areas in each sample before treatment. (B) Pearson correlation coefficient was used to compare the correlation of FoxP3+, CD3 + CD8+, CD3 + FoxP3+, CD15+, PD-1+, CD8 + PD-1 + and PD-L1 + cell infiltration density in the stroma and tumor area. Cell density is represented as the number of cells per area (mm2) analyzed. [file 12885_2024_12406_MOESM1_ESM.jpg]

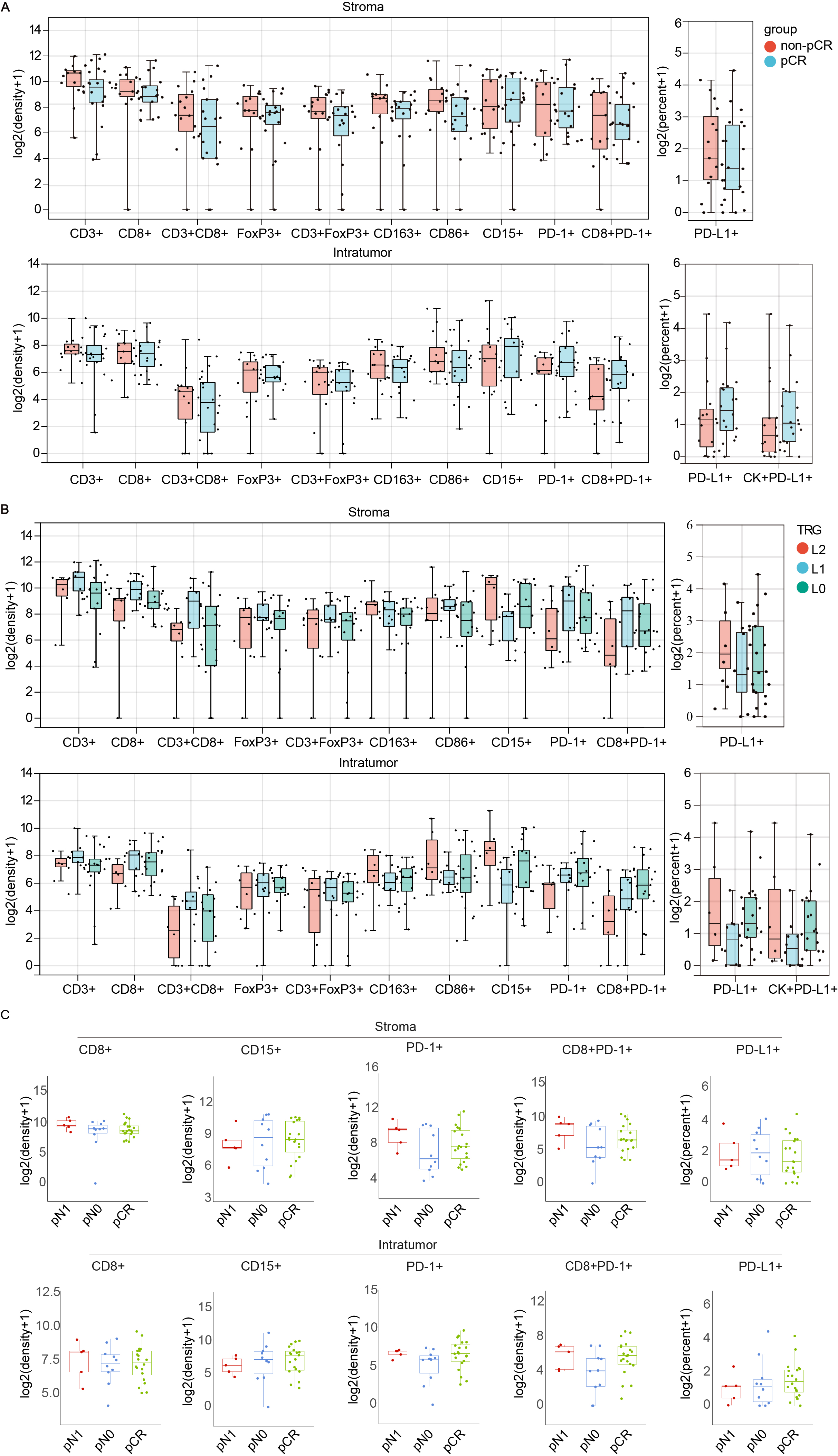

Supplement: Supplementary file 2 — Supplementary Material 2: Figure S2. Comparison of immune cells infiltration density in pretreatment samples in different pathological grades. (A) The infiltration of immune cells in different regions were compared among pCR and non-pCR. (B) The infiltration of immune cells in different regions were compared among TRG0, TRG1 and TRG2. (C) The infiltration of CD8+, CD15+, PD-1, CD8 + PD-1 + and PD-L1 + cells in different regions were compared among pCR, pN0 and pN1. (Statistical significance was determined using a T-test.) [file 12885_2024_12406_MOESM2_ESM.jpg]

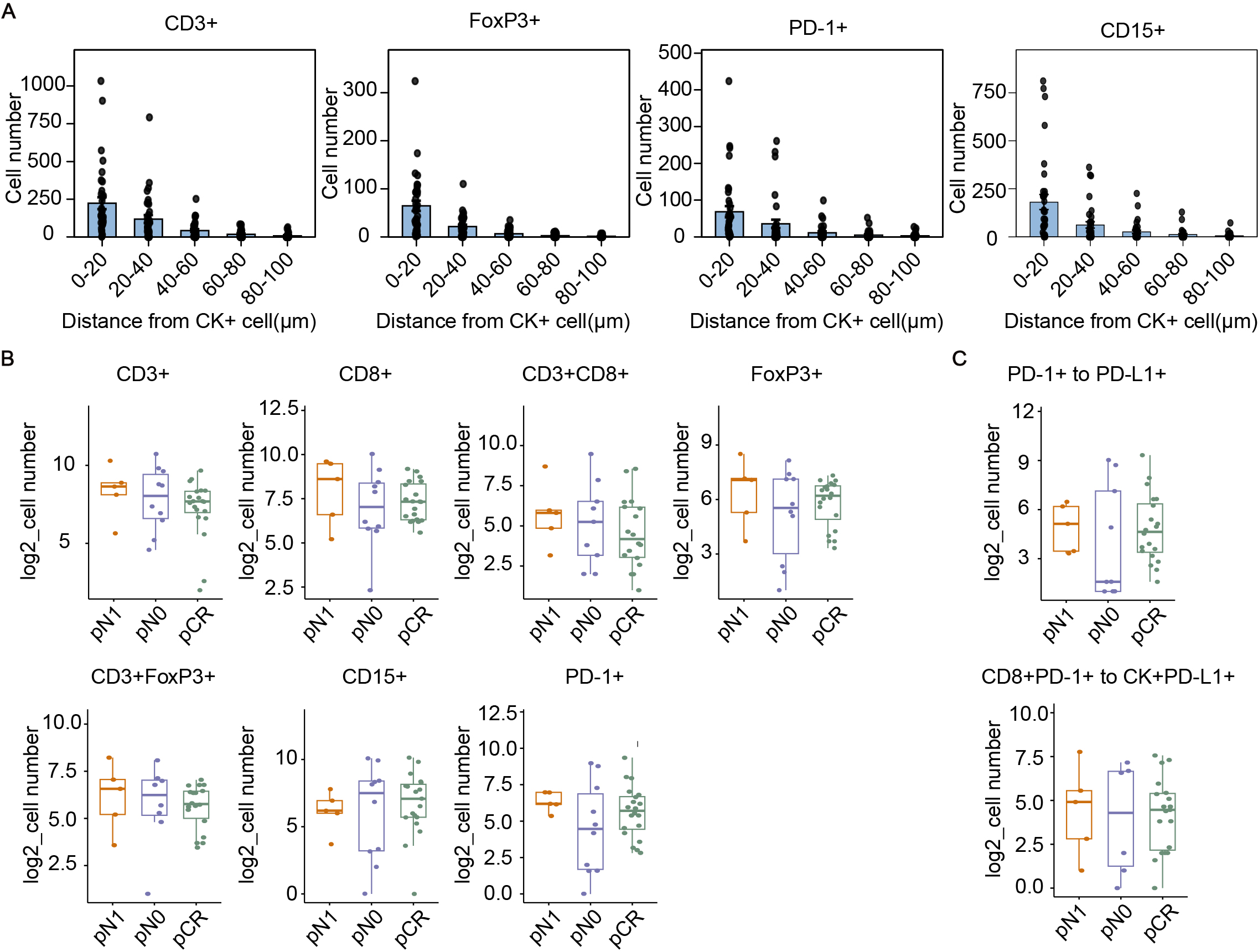

Supplement: Supplementary file 3 — Supplementary Material 3: Figure S3. Spatial distribution of immune cells in pretreatment samples. (A) CD3+, FoxP3+, PD-1 + and CD15 + cells localization around the tumor areas and they infiltration into the tumor are expressed by the distance from CK + cells in µm. (B) Comparison of the quantity of infiltrating CD3+, CD8+, CD3 + CD8+, FoxP3+, CD3 + FoxP3+, CD15 + and PD-1 + cells within a 40 µm proximity to tumor cells in pre-treatment pCR, pN0, and pN1 ESCC tissues. (C) Comparison of the quantity of infiltrating PD-1 + T lymphocytes within a 40 µm proximity to PD-L1 + cells and CD8 + PD-1 + T lymphocytes within a 40 µm proximity to CK + PD-L1 + tumor cells in pre-treatment pCR, pN0, and pN1 ESCC tissues. (Statistical significance was determined using a T-test.) [file 12885_2024_12406_MOESM3_ESM.jpg]

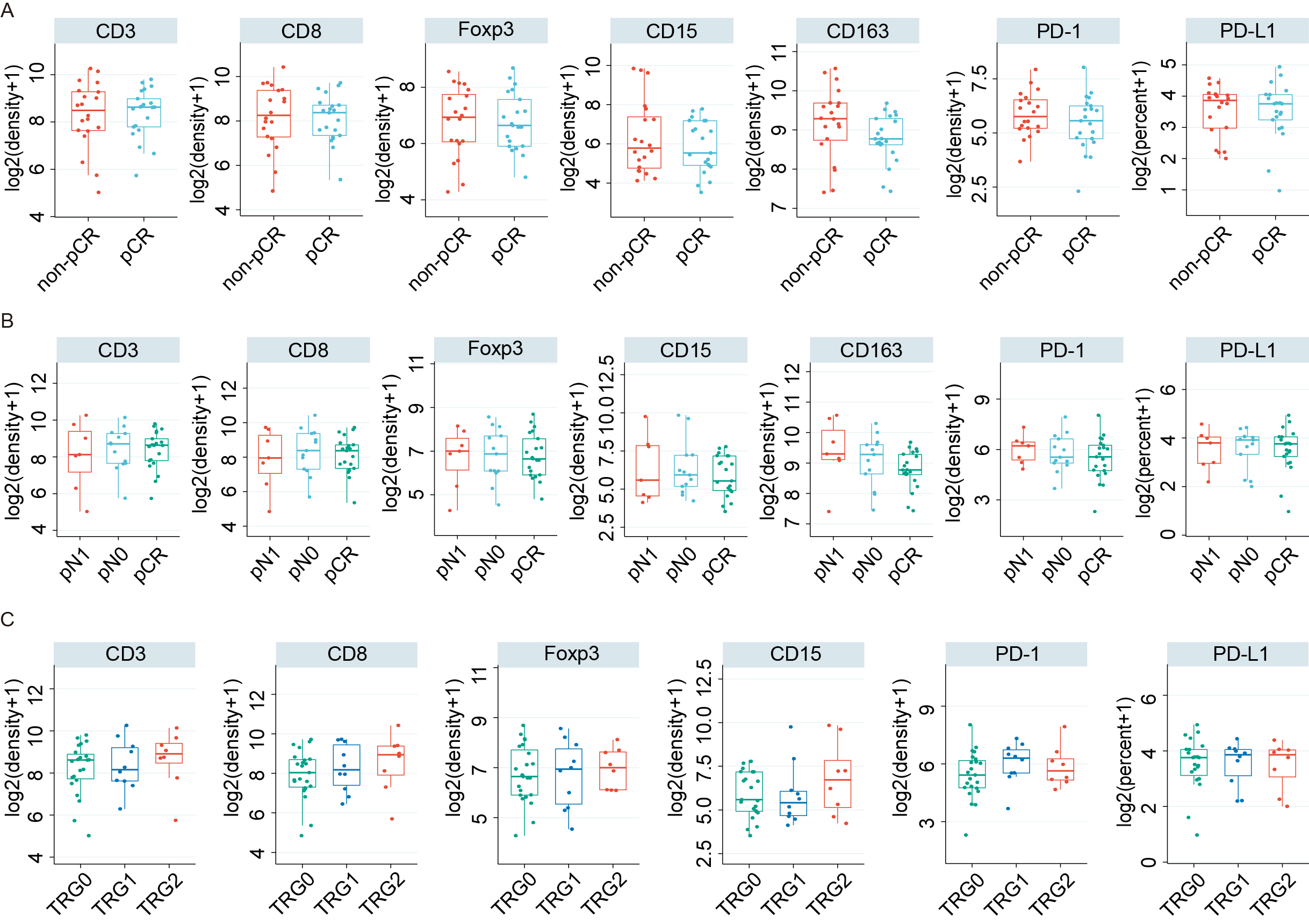

Supplement: Supplementary file 4 — Supplementary Material 4: Figure S4. Comparison of infiltration density of immune cells in post-treatment samples in different pathological grades. (A) The infiltration of immune cells in different regions were compared among pCR and non-pCR. (B) The infiltration of immune cells in different regions were compared among pCR, pN0 and pN1. (C) The infiltration of immune cells in different regions were compared among TRG0, TRG1 and TRG2. (Statistical significance was determined using a T-test.) [file 12885_2024_12406_MOESM4_ESM.jpg]

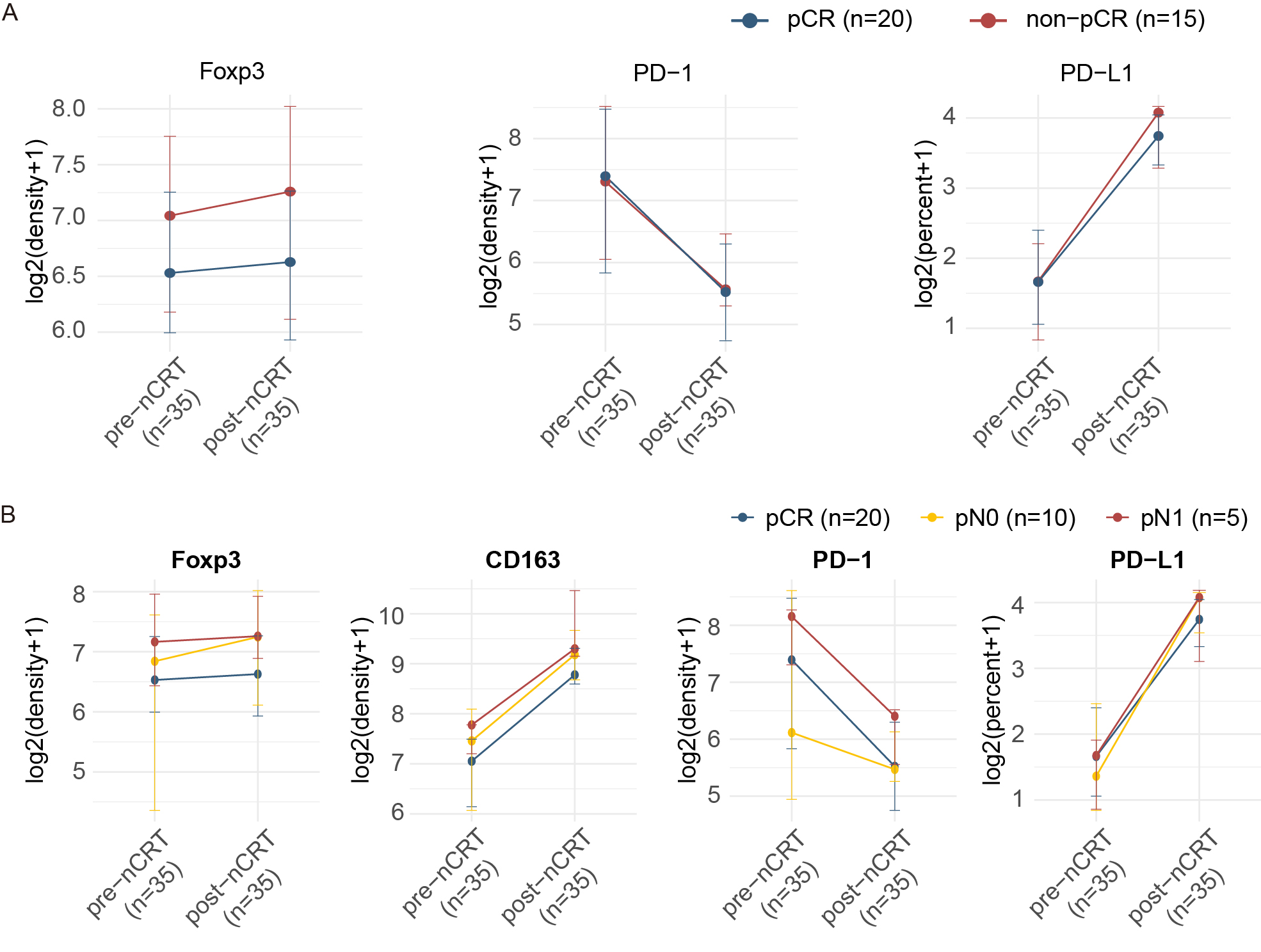

Supplement: Supplementary file 5 — Supplementary Material 5: Figure S5. Changes in the immune landscape during treatment. (A) Densities of FoxP3+, PD-1 + and PD-L1 + cells before and after treatment between the pCR and non-pCR samples. (B) Densities of FoxP3+, CD163+, PD-1 + and PD-L1 + cells before and after treatment among the pCR, pN0 and pN1 samples. Points represent median values, whereas whiskers show the upper and lower quantiles. [file 12885_2024_12406_MOESM5_ESM.jpg]
